# AppendiXNet: Deep Learning for Diagnosis of Appendicitis from A Small Dataset of CT Exams Using Video Pretraining

**DOI:** 10.1038/s41598-020-61055-6

**Published:** 2020-03-03

**Authors:** Pranav Rajpurkar, Allison Park, Jeremy Irvin, Chris Chute, Michael Bereket, Domenico Mastrodicasa, Curtis P. Langlotz, Matthew P. Lungren, Andrew Y. Ng, Bhavik N. Patel

**Affiliations:** 1 Stanford University Department of Computer Science, Stanford, USA; 2 Stanford University Department of Radiology, Stanford, USA; 3 Stanford University AIMI Center, Stanford, USA

**Keywords:** Medical imaging, Medical imaging, Computed tomography, Computed tomography

## Abstract

The development of deep learning algorithms for complex tasks in digital medicine has relied on the availability of large labeled training datasets, usually containing hundreds of thousands of examples. The purpose of this study was to develop a 3D deep learning model, AppendiXNet, to detect appendicitis, one of the most common life-threatening abdominal emergencies, using a small training dataset of less than 500 training CT exams. We explored whether pretraining the model on a large collection of natural videos would improve the performance of the model over training the model from scratch. AppendiXNet was pretrained on a large collection of YouTube videos called Kinetics, consisting of approximately 500,000 video clips and annotated for one of 600 human action classes, and then fine-tuned on a small dataset of 438 CT scans annotated for appendicitis. We found that pretraining the 3D model on natural videos significantly improved the performance of the model from an AUC of 0.724 (95% CI 0.625, 0.823) to 0.810 (95% CI 0.725, 0.895). The application of deep learning to detect abnormalities on CT examinations using video pretraining could generalize effectively to other challenging cross-sectional medical imaging tasks when training data is limited.

## Introduction

Appendicitis is one of the most common life-threatening abdominal emergencies, with lifetime risk of ranging from 5–10% and an annual incidence of 90–140 per 100,000 individuals^1–3^. Treatment, via appendectomy, remains the most frequently performed surgical intervention in the world^4^. Computed tomography (CT) imaging of the abdomen is the primary imaging modality to make the diagnosis of appendicitis, exclude other etiologies of acute abdominal pain, and allow for surgical planning and intervention. Because a delay in the time between CT imaging diagnosis and surgical appendectomy can worsen patient outcomes^5–9^, there is increasing pressure on hospital systems to provide 24-7 access to advanced imaging and to ensure that the results of urgent findings, such as appendicitis, are rapidly and accurately communicated to the referring physician^10,11^. However, providing rapid and accurate diagnostic imaging is increasingly difficult to sustain for many medical systems and radiology groups as utilization has rapidly expanded; for example, the CT utilization rate in the emergency room increased from 41 per 1000 in 2000 to 74 per 1000 in 2010, with abdominal CTs accounting for over half of this volume^12,13^. Development of automated systems could potentially help improve diagnostic accuracy and reduce time to diagnosis, thereby improving the quality and efficiency of patient care.

Recent advancements in deep learning have enabled algorithms to automate a wide variety of medical tasks^14–20^. A key aspect for the success of deep learning models on these tasks is the availability of large labeled datasets of medical images^21^, usually containing hundreds of thousands of examples. However, it is challenging to curate large labeled medical imaging datasets of that scale. Pretraining^22^ can mitigate this problem, but pretraining employs large datasets of natural images such as ImageNet. Such training has been effective for 2D medical imaging tasks (where datasets are comparatively smaller), but does not easily apply to 3D data produced by cross sectional imaging devices. The use of large datasets of natural videos as pretraining for 3D medical imaging tasks remains unexplored.

In this study, we developed a deep learning model capable of detecting appendicitis on abdominal CT using a small dataset of 438 CT examinations. We explored whether pretraining the model on a large collection of natural videos would improve the performance of the model over training the model from scratch. Successful application of deep learning to detect abnormalities on CT examinations using video pretraining could generalize effectively to other cross-sectional medical imaging deep learning applications where training data is limited and the task is challenging.

## Methods

This retrospective, single-center, Health Insurance Portability and Accountability Act-compliant study was approved by the Institutional Review Board of Stanford University, and a waiver of informed consent was obtained.

### Data

CT exams of the abdomen and pelvis performed at the Stanford University Medical Center were reviewed. All exams were performed on a GE Medical Systems scanner, with slice thicknesses of 0.625 mm, 1.25 mm, or 2.5 mm, performed with 16 detector row CT scanner (LightSpeed VCT; GE Healthcare, Waukesha, WI). Reconstructed 1.25 mm axial slices were used to develop and validate the model. Patients were administered intravenous iodinated contrast material (Isovue 370; Bracco Diagnostics, Monroe Township, NJ) using an injection volume of 1.5 mL/kilogram of body weight and at a rate of 2.5–3.5 mL/s. CT images were acquired 70 seconds after the injection of contrast. CT exams were manually reviewed by a board-certified subspecialty-trained abdominal radiologist with 6 years of experience. Adult patients who presented to the emergency room with abdominal pain and who subsequently underwent a contrast-enhanced CT exam were included. Pregnant patients, patients with prior appendectomies, and patients who underwent unenhanced exams were excluded. The axial series of each exam was used.

**Figure 1:**
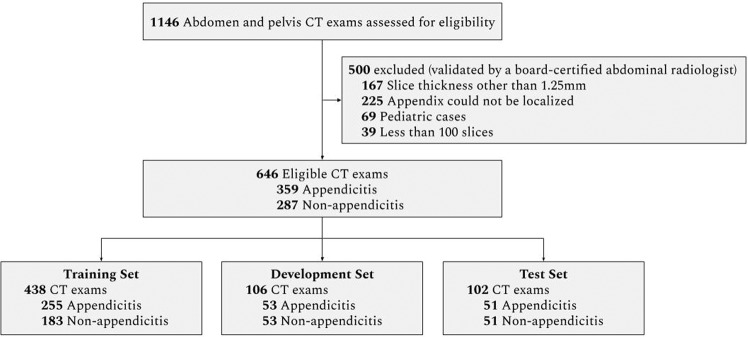
Data Set Selection Flow Diagram.

The exams were split into a training set used to train the model, a development set used for model selection, and a test set to evaluate the final performance of the model. Using stratified random sampling, the development and test sets were formed to include approximately 50% appendicitis exams and 50% non-appendicitis exams. There was no patient overlap between the different sets. Figure 1 shows the dataset selection flow diagram and Table 1 contains pathology and patient demographics for each set.

**Table 1:** Demographic information for the training, development, and test datasets.

Statistic	Training	Development	Test
Studies, N	438	106	102
Patients, N	435	105	102
Female, N (%)	253 (58.2)	64 (61.0)	64 (62.7)
Age, Y mean (SD)	38.2 (15.6)	39.2 (17.3)	38.4 (15.7)
Abnormal, N (%)	255 (58.2)	53 (50.0)	51 (50.0)

### Reference standard

The reference standard for all examinations was determined by a board-certified, fellowship-trained abdominal radiologist with 6 years of experience, who determined the presence and absence of appendicitis by review of the original radiology report, all of the DICOM series, the original report, and the clinical history. For the test set ground truth, exams were given a label of appendicitis, if all of the following criteria were met: (a) the patient’s radiology report at the time of exam was rendered a diagnosis of acute appendicitis; (b) appendicitis was confirmed upon manual review of the CT scan by the aforementioned radiologist; and (c) the patient underwent appendectomy and inflammation of the appendix was confirmed by the pathology report. For negative labels, all of the following criteria had to be met: (a) the patient was scanned for abdominal pain and a clinical concern for appendicitis; (b) the radiology report at the time of exam did not render a diagnosis of acute appendicitis; (c) a normal appendix was confirmed upon manual review of the CT scan by the aforementioned radiologist; and (d) the patient did not undergo appendectomy or image-guided percutaneous drainage as a result of the index CT and was discharged or treated for another unrelated diagnosis (e.g. renal stone).

### Model development

**Figure 2:**
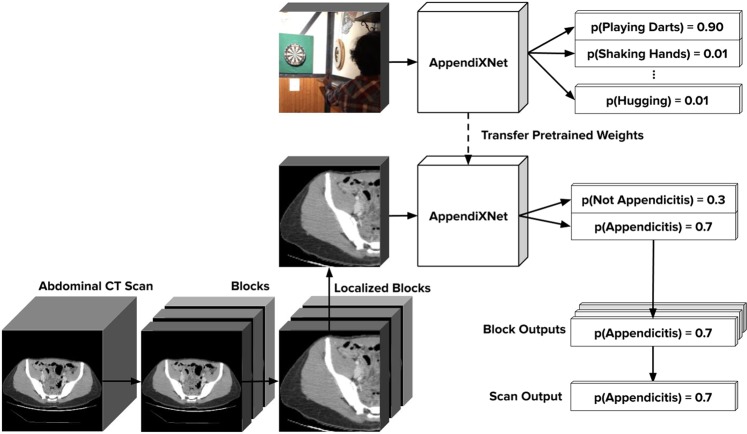
AppendiXNet Training: AppendiXNet was first pretrained on Kinetics, a large collection of labeled YouTube videos. After pretraining AppendiXNet, the network was fine-tuned on the appendicitis task after replacing the final fully connected layer with one which produces a single output.

We developed AppendiXNet, an 18-layer 3D convolutional neural network^23^ for classification of appendicitis from CT exams. A convolutional neural network (CNN) is a machine learning model designed to process image data. 3D convolutional neural networks are particularly well suited to handle sequences of images such as a video or a series of cross-sectional images^24^. AppendiXNet takes as input a CT scan and outputs a probability indicating the presence or absence of appendicitis in that scan. To process the scan under the memory constraints of the GPU, AppendiXNet takes in groups of 8-slices and produces a single probability for the group. Each non-overlapping group of 8-slices is input to the model to produce probabilities over the whole scan. When there are not enough slices to fill the group, the group is padded with slices containing all zeros. The probabilities for all of the groups within a scan are combined by first applying a rolling mean with a window size of 3 to the group probabilities, and then computing the maximum average probability over the resulting means as the probability of appendicitis in the entire scan. For each of the exams, a board-certified fellowship-trained practicing abdominal radiologist identified the slices of the axial series that contained the appendix (both normal and abnormal). We integrated these annotations into our training procedure. This procedure is diagrammed in Fig. 2. The complete training procedure is detailed in the Supplementary Information.

AppendiXNet was first pretrained on a large collection of YouTube videos called Kinetics. Kinetics consists of approximately 500,000 video clips, each approximately 10 seconds long. Each of the video clips has been annotated for one of 600 human action classes such as ‘playing instruments’, ‘shaking hands and hugging’ etc. Similar to transfer learning for image problems, after pretraining AppendiXNet, the network was fine-tuned on the appendicitis task after replacing the final fully connected layer with one which produces a single output. We compared the performance of AppendiXNet with and without pretraining on Kinetics.

#### Model architecture

AppendiXNet uses a 3D residual network (3D ResNet) architecture^25^, which consists of 4 stacked residual blocks (after an initial 7 × 7 × 7 convolutional layer and 3 × 3 × 3 max pooling layer) with the same structure across stages: a 3 × 3 × 3 convolutional layer, a 3D batch normalization layer, a ReLU nonlinearity, another 3 × 3 × 3 convolutional layer and 3D batch normalization layer, a downsampling (strided average pooling) layer, then a residual connection followed by a final ReLU nonlinearity. After the 4 blocks, global average pooling is applied, followed by a fully connected layer with a single output. A sigmoid nonlinearity is applied to the output of the fully connected layer to obtain the final probability.

We compared AppendiXNet to a variety of other convolutional neural network architectures including (1) 18-layer and 34-layer 2D ResNet architectures^26^ where the output of the fully connected layers on each slice are combined with an average, (2) Long-term Recurrent Convolutional Networks (LRCN)^27^ with 18-layer and 34-layer 2D ResNet backbones, and (3) a 50-layer squeeze and excitation 3D ResNeXt architecture^28^. The 3D models were pretrained on Kinetics^29^ and the 2D models were pretrained on ImageNet^30^. To minimize the chances of overfitting to the test set, we ran these comparisons on the development set.

#### Model interpretation

Model predictions were interpreted through the use of Gradient-weighted Class Activation Mappings (Grad-CAMs) extended to the 3D setting^31^. Grad-CAMs are visual explanations of model predictions, and can be visualized as heat maps overlaying each slice. To generate the Grad-CAM for an input, the feature maps output by the final convolutional layer of AppendiXNet were combined using a weighted average, where the weights are the gradients flowing into the final convolutional layer. The resulting Grad-CAM was upscaled to the dimensions of the input using quadratic interpolation and then overlaid to highlight the salient regions of the input used in the classification as a heat map. In order to visualize the 3D Grad-CAM, we plot each slice of the input with the corresponding slice of the Grad-CAM overlaid.

### Statistical methods

For the binary task of determining whether a scan contained appendicitis, the area under the receiver operating characteristic curve (AUC), sensitivity, specificity, and accuracy were used to assess the performance of the model on the holdout test set. The probabilities output by the models were binarized by finding the cutoff probability which led to a sensitivity of 0.9 on the development set; the sensitivity threshold of 0.9 was chosen for the models based on clinical appropriateness for the diagnostic task of evaluating the appendix on CT^32^. A prediction was considered positive if the model predicted a probability higher than the model’s cutoff probability, and negative otherwise. Confidence intervals at 95% were constructed for the AUC using DeLong’s method, and using the Wilson method for assessing the variability in the estimates for sensitivity, specificity, and accuracy.

To assess improvements in performance with pretraining, the difference in AUCs was reported, and tested for statistically significant improvement using the DeLong method; statistical significance was assessed at the 0.05 level so a p-value < 0.05 indicates statistical significance. This retrospective, Health Insurance Portability and Accountability Act-compliant study was approved by the Institutional Review Board of Stanford University.

## Results

Of 646 CT examinations from 642 unique patients, 359 (55.6%) exams showed appendicitis and 287 exams showed no appendicitis. There were no systematic differences in protocols or slice thicknesses between the appendicitis and non-appendicitis exams. The number of images per exam ranged from 101 to 778 (mean: 330.2, std: 97.7), with a total of 213,335 images in the full dataset. The training set consisted of 438 exams (255 appendicitis and 183 non-appendicitis from 435 patients), the development set consisted of 106 exams (53 appendicitis and 53 non-appendicitis from 105 patients), and the test set consisted of 102 exams (51 appendicitis and 51 non-appendicitis from 102 patients). Figure 1 shows the dataset selection flow diagram, and Table 1 details demographic information for the training, development, and test datasets.

On the holdout test set, AppendiXNet achieved an AUC of 0.810 (95% CI 0.725, 0.895); at its high-sensitivity operating point, AppendiXNet achieved a sensitivity of 0.784 (95% CI 0.654, 0.875), specificity of 0.667 (95% CI 0.530, 0.780), and accuracy of 0.725 (95% CI 0.632, 0.803). AppendiXNet took 24 hours to train, and 5 minutes to make predictions on the test set.

**Figure 3:**
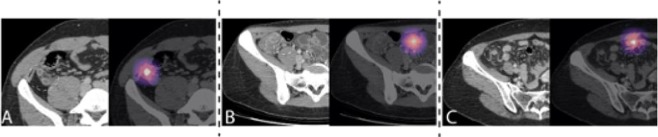
Interpreting AppendiXNet Predictions. CT (grayscale; left) and corresponding gradient-weighted class activation map (Grad-CAM) (colored; right) are provided. (**A**) Example of true positive. CT image (left) shows acute appendicitis (dashed circle). The deep learning model correctly predicted appendicitis as seen on the corresponding Grad-CAM. (**B**) Example of false positive. CT image (left) shows a normal appendix (dashed circle). The deep learning model incorrectly predicted appendicitis as seen on the corresponding Grad-CAM where it focused on a loop of distal ileum which was used for a false positive prediction. (**C**) Example of false negative. CT image (left) shows early acute appendicitis (dashed circle). The deep learning model incorrectly provided a low predicted probability for appendicitis as seen on the corresponding Grad-CAM where it focused on a loop of distal lieum.

Pretraining AppendiXNet on a large dataset of natural videos produced a statistically significant increase in the AUC, with a mean increase of 0.086 (p-value = 0.025), from an AUC of 0.724 (95% CI 0.625, 0.823) without pretraining to an AUC of 0.810 (0.725, 0.895) with pretraining. At its high-sensitivity operating point, AppendiXNet achieved a sensitivity of 0.784 (95% CI 0.654, 0.875), specificity of 0.353 (95% CI 0.236, 0.490), and accuracy of 0.569 (95% CI 0.472, 0.661). The performance of AppendiXNet on the holdout test set is summarized in Table 2. Examples of Grad-CAM heatmaps produced by AppendiXNet are shown in Fig. 3. The results of using different training strategies on the development set are shown in Supplementary Table 2.

**Table 2:** Performance measures of AppendiXNet on the independent test set with and without pretraining.

Model	AUC	Specificity	Sensitivity	Accuracy
Pretrained on video images	0.810 (0.725, 0.895)	0.667 (0.530, 0.780)	0.784 (0.654, 0.875)	0.725 (0.632, 0.803)
Not pretrained on video images	0.724 (0.625, 0.823)	0.353 (0.236, 0.490)	0.784 (0.654, 0.875)	0.569 (0.472, 0.661)

Although we did not test for significance, the increase in AUC with pretraining was observed on the development set across all model architectures. The 2D ResNet-18-based model achieved 0.704 (0.605, 0.803) without pretraining and 0.763 (0.672, 0.854) with pretraining. The LRCN ResNet-18 model achieved an AUC of 0.488 (0.376, 0.600) without pretraining and 0.787 (0.699, 0.875) with pretraining. The SE-ResNeXt-50 model achieved an AUC of 0.503 (0.391, 0.614) without pretraining and 0.721 (0.625, 0.817) with pretraining. AppendiXNet with pretraining outperformed all other models with pretraining. The performance of the different model architectures on the development set is summarized in Table 3.

**Table 3:** Area under the receiver operating characteristic curve (AUC) of different models on the development set with and without pretraining.

Training Strategy	AUC (95% CI)	
	Not Pretrained	Pretrained
AppendiXNet	0.743 (0.649, 0.837)	0.826 (0.742, 0.909)
Average of 2D ResNet-18	0.704 (0.605, 0.803)	0.763 (0.672, 0.854)
Average of 2D ResNet-34	0.740 (0.644, 0.835)	0.802 (0.715, 0.888)
LRCN ResNet-18	0.706 (0.605, 0.806)	0.778 (0.690, 0.867)
LRCN ResNet-34	0.488 (0.376, 0.600)	0.787 (0.699, 0.875)
SE-ResNeXt-50	0.503 (0.391, 0.614)	0.721 (0.625, 0.817)

## Discussion

We developed a three-dimensional (3D) convolutional neural network (CNN), AppendiXNet, for automated detection of acute appendicitis on contrast-enhanced CT of the abdomen and pelvis. We found that the network achieved high discriminative performance (AUC = 0.810 (95% CI 0.725, 0.895)) using a small training dataset of 438 CT examinations, with a significant increase in performance (p = 0.025) attributed to video-pretraining.

While automated segmentation tasks involving CT of the abdomen have been described^33–35^, few deep learning models for automated classification tasks involving abdominopelvic CTs have been reported^36^. In particular, no automated detection tasks involving clinically emergent focal abnormalities such as appendicitis have been reported. The paucity of published diagnostic models for abdominal CT may relate to the difficulty of the task. For example, a normal appendix is a relatively small structure compared to the entire image, measuring 6 mm^37^ on a 2D axial CT image which measures approximately 32 cm in transverse dimension for the average human body^38^. Moreover, the appendix may only be present on a few images of an entire exam that can comprise up to several hundred images depending on the slice thickness leading to class imbalance. Other contributory challenges include overlap in CT appearance of normal and abnormal appendices^39^, varied location within the body^40^, the myriad image manifestations of an inflamed appendix, and non-visualization of the normal appendix in 43–58% of CT examinations^41^. The performance of our 3D CNN for automated detection of acute appendicitis with high diagnostic performance of 0.810 (95% CI 0.725, 0.895 is comparable to previously published 3D CNN models for automated detection of emergent findings on CT^42^.

We hypothesized that we could improve the performance of our deep learning model to detect appendicitis by pretraining the model on a dataset of natural videos. We pretrained our model on Kinetics, a collection of over 500,000 YouTube videos and found that the diagnostic performance of the model increased from 0.724 (95% CI 0.625, 0.823) without pretraining to 0.810 (95% CI 0.725, 0.895) after pretraining on a dataset of only 438 CT examinations. We also found that video-pretraining increased the performance of other 3D architectures, and image-pretraining increased the performance of 2D architectures. Interpretation of cross-sectional medical images often requires 3D context, involving continuous scrolling of contiguous 2D slices of a patient’s scan. Thus, the presence of abnormalities or pathologies become apparent using spatio-temporally related information from multiple slices. Moreover, visual representations necessary for complex spatiotemporal understanding tasks may not be adequately learned using static images; instead feature representations can be learned using videos, resulting in improved model performance^43,44^. Our work shows the utility of pretraining with videos with a small clinical dataset size, and could generalize to other challenging cross-sectional medical imaging applications with limited data sets.

This work has several limitations that merit consideration in addition to those associated with a retrospective study. First, we had a small training dataset, and did not investigate the effect of video pretraining as a function of the training data size. As investigated in other medical imaging tasks^45^, we expect that the effect of pretraining will diminish as the target data size increases. Second, we investigated the effect of pretraining model on the Kinetics dataset, but pretraining on a medical imaging dataset more similar to CT scans may lead to better performance and would be worth exploring in future work^46^. Third, our clinical training data was from a single center, performed on devices from a single vendor using our standard institutional technique for image acquisition, limiting generalizability. Fourth, our model did not distinguish among different forms of acute appendicitis that would affect management; that is, it did not distinguish between uncomplicated and complicated (i.e. perforated) appendicitis.

In conclusion, we developed a 3D model for detecting acute appendicitis on CT exams on a small training dataset of CT examinations using video-pretraining. Our work highlights a novel model development technique that employs video pretraining to compensate for a small dataset and may be utilized in future deep learning studies involving medical images.

## Supplementary information

Supplemental Material.
