# Supplementary material for: AppendiXNet: Deep Learning for Diagnosis of Appendicitis from A Small Dataset of CT Exams Using Video Pretraining
Source: Sci Rep. 2020 Mar 3;10:3958. doi: 10.1038/s41598-020-61055-6 (PMC7054445; doi:10.1038/s41598-020-61055-6)
Supplement: Supplementary file 1 — Supplemental Material. [file 41598_2020_61055_MOESM1_ESM.docx]

**Supplementary Material**

**AppendiXNet: Deep Learning for Diagnosis of Appendicitis from A Small Dataset of CT Exams Using Video Pretraining**

Pranav Rajpurkar, MS^1,†^, Allison Park, MS^1,†^, Jeremy Irvin, MS^1,†^, Chris Chute, MS^1^, Michael Bereket, MS^1^, Domenico Mastrodicasa, MD^2^, Curtis P. Langlotz MD, PhD^3^, Matthew P. Lungren, MD, MPH^3^, Andrew Y. Ng, PhD^1,‡^, Bhavik N. Patel, MD, MBA^2,3,‡,*^

Affiliations

1 Stanford University Department of Computer Science

2 Stanford University Department of Radiology

3 Stanford University AIMI Center

† These authors contributed equally to this work

‡ These authors contributed equally to this work

* Corresponding Author

*Training Procedure*

AppendiXNet was trained on groups of 8 consecutive slices in the axial plane; groups of 8 slides were chosen to balance the tradeoff between necessary context to identify the appendix and memory constraints of the GPU. Each group of slices was treated as a positive example for appendicitis if and only if the group of slices contained the appendix and the exam was positive for appendicitis. Before being fed into the network, groups of slices were clipped to between [-360, 440] Hounsfield Units, corresponding to the soft tissue window for the abdomen (window width 400, level 40), and each slice scaled to 224x224 pixels. Before the forward pass, each slice has the single channel dimension replicated three times for CT inputs to simulate an RGB input used to train with natural videos. We experimented with data augmentations such as flipping the scans vertically and horizontally, and randomly cropping parts of the scan, but did not include them as part of our final model since we did not find that they produced significant improvements.

All model variants were optimized on the binary cross-entropy loss using stochastic gradient descent (SGD) with momentum to update the model’s parameters. The learning rate was tuned for each model and strategy by selecting the initial learning rate among 5e-4, 5e-5, and 5e-6 which led to the highest AUC on the development set. The optimal learning rate for AppendiXNet was 5e-6. For each model and training strategy, the momentum parameter was set to 0.9 and the dampening was set to 0. Every 60 scans, the model was evaluated on the development set and the model was saved based on the best development loss. Additionally the learning was decayed by 0.1 when the development loss stopped decreasing. For regularization, L2 weight decay of 0.001 was added to the loss for all trainable parameters. The final model for each training strategy and model variant was an ensemble of 3 models trained using the training strategy with different random weight initialization. All models were trained on NVIDIA GeForce GTX 1070 GPUs using the PyTorch library v0.4.1 using using a batch size of 7 examples.

*Model Architectures*

In addition to the architectures included in the main text, we also tried experimenting with many other architectures with pretrained weights (121-layer 3D DenseNet, 34-layer, 50-layer, and 110-layer 3D ResNets, 101-layer 3D ResNeXt, 50-layer 2D ResNet whose outputs were combined with an average, and an LRCN with a 50-layer 2D ResNet backbone), but these experiments could not be run due to memory constraints of the GPU.

*Training Strategies*

We investigated the effect of using different training strategies on the performance of AppendiXNet on the development set. The different strategies are described below, and the performance of the model on the development set under each strategy is reported in Supplementary Table 1.

*Oversampling*

When training AppendiXNet, we oversampled slices containing the appendix for both normal and abnormal examinations. Groups of slices containing the appendix⁠—defined as having at least 8 slices marked to include the appendix⁠—were forcefully sampled with a probability of *p*; otherwise groups of slices were randomly sampled. We reported the performance of the model on the test set with different values of *p*, where *p=0* indicates random sampling in Supplementary Table 1.

*Localization*

Before axial CT images were fed into the model, we automatically cropped the images around the locations of the appendix in the x-y plane. Using the software ITK-SNAP, for a subset of 106 appendicitis exams, a board-certified radiologist annotated a bounding box around the appendicitis. We used these annotations to develop a rule to localize the appendix in the x-y plane: we first extracted the contour of the body region within the scan, and then extracted the top left quadrant of the contour. We tweaked this localization rule by measuring the overlap between the field of view and the bounding boxes around the appendix until we could verify that the localized scan completely included the appendix in view for all annotated training scans. We report the performance of the model with and without this localization in Supplementary Table 1.

**Supplementary Tables**

| **Training Strategy** | **AUC (95% CI)** |
| --- | --- |
| AppendiXNet, with localization,  and w/ pretraining  oversampling w/ p(oversample)=0.7, | 0.826 (0.742, 0.909) |
| Without localization | 0.686 (0.584, 0.789) |
| Without pretraining | 0.743 (0.649, 0.837) |
| Without oversampling | 0.805 (0.723, 0.888) |
| Oversampling w/ p(oversample)=0.3 | 0.820 (0.738, 0.901) |
| Oversampling w/ p(oversample)=0.5 | 0.828 (0.750, 0.906) |
| Oversampling w/ p(oversample)=0.9 | 0.822 (0.739, 0.905) |

**Supplementary Table 1.** Area under the receiver operating characteristic curve (AUC) of different modeling strategies on the development set.
